# High-risk coronary plaque of sudden cardiac death victims: postmortem CT angiographic features and histopathologic findings

**DOI:** 10.1007/s00414-024-03228-w

**Published:** 2024-04-10

**Authors:** Katarzyna Michaud, David C Rotzinger, Mohamed Faouzi, Silke Grabherr, Salah D Qanadli, Allard C van der Wal, Virginie Magnin

**Affiliations:** 1 University Center of Legal Medicine Lausanne - Geneva, Lausanne University Hospital and University of Lausanne, Chemin de la Vulliette 4, Lausanne 25, CH - 1000 Switzerland; 2 Department of Diagnostic and Interventional Radiology, Lausanne University Hospital and University of Lausanne, Lausanne, Switzerland; 3 Center for Primary Care and Public Health, Division of Biostatistics, Lausanne, Switzerland; 4 Faculty of Biology and Medicine, University of Lausanne, Lausanne, Switzerland; 5 Riviera-Chablais Hospital, Rennaz, 1847 Switzerland; 6 Amsterdam UMC, Academic Medical Center, Amsterdam, The Netherlands; 7 Maastricht University Medical Center (MUMC), Maastricht, The Netherlands

**Keywords:** Postmortem angiography, Computed tomography, Coronary plaque, High-risk plaque, Sudden cardiac death, Coronary thrombosis

## Abstract

High-risk coronary plaques (HRP) are characterized in clinical radiological imaging by the presence of low plaque attenuation, a napkin-ring sign (NRS), spotty calcifications (SC) and a positive remodeling index (RI). To evaluate if these signs are detectable in postmortem imaging by a multi-phase postmortem CT angiography (MPMCTA), a retrospective study of a series of autopsy well-documented coronary plaques related to sudden cardiac death (SCD) was performed. Then correlations between histological and radiological findings were described. Fourty SCD cases due to acute coronary syndrome based on clinical history and confirmed at autopsy were selected (28 men and 12 women, age 53.3 ± 10.9). The culprit lesion was mainly situated in the proximal segments of coronary arteries, in the right coronary artery in 23 cases (57.5%), the left anterior descending artery in 13 cases (32.5%), the circumflex artery in 3 cases (7.5%) and in one case in the left main stem. MPMCTA showed a positive RI (≥ 1.1) in 75% of cases with a mean RI 1.39 ± 0.71. RI values were lower in cases with fibrotic plaques. NRS was observed in 40% of cases, low attenuation plaque in 46.3%, and SC in 48.7% of cases. There were significant correlations of the radiological presence of NRS for fibrolipid composition of the plaque (p-value 0.007), severe intraplaque inflammation (p-value 0.017), severe adventitial inflammation (p-value 0.021) and an increased vasa vasorum (p-value 0.012). A significant correlation (p-value 0.002) was observed between the presence of SC at radiological examination and the presence of punctuate/fragmented calcification at histology. In addition, in 58.3% of cases, plaque enhancement was observed, which correlated with plaque inflammation and the fibrolipid composition of the plaque. The coronary artery calcium score was 314 (± 455). There was a poor agreement between stenosis of the lumen at histology versus radiology. Our study shows that the various radiological signs of HRP can be detected in all plaques by MPMCTA, but individually only to a variable extent; plaque enhancement appeared as a new sign of vulnerability. In the postmortem approach, these radiological markers of HRP, should always be applied in combination, which can be useful for developing a predictive model for diagnosing coronary SCD.

## Background

Ischemic heart disease (IHD) resulting from atherosclerotic coronary artery disease (CAD) remains the most frequent cause of death in Western countries [1]. Acute manifestations of CAD, acute coronary syndrome, relate primarily to acute thrombotic lumen occlusion initiated by disruption of a plaque by either fibrous cap ruptures or surface erosions. Pathological analysis of these acute coronary culprit plaques at autopsy has revealed several tissue characteristics of lesions that are particularly prone to disruptions, the so-called ‘high-risk plaques (HRP), formerly referred to as vulnerable plaques (VP). Most distinctive features are a thin-cap fibroatheroma (TCFA), large lipid atheroma, significant plaque inflammation, plaque hemorrhage, and occurrence of spotty calcifications [2, 3].

In *clinical* practice, coronary computed tomography angiography (CCTA) is an established and non-invasive imaging modality that can evaluate CAD’s presence, severity, and distribution. In the setting of acute chest pain, growing evidence supports the use of CCTA with high reported accuracy for obstructive disease, at lower cost, and with less radiation than nuclear imaging testing [4]. It allows not only the detection of coronary artery stenosis but also quantitative analysis of stenosis rate or total occlusive disease and qualitative assessment of parameters of plaque morphology [5–7]. Radiological features specific to high-risk plaques (HRP) include the presence of lowattenuation plaque, napkin-ring sign (NRS), spotty calcifications (SC), and positive remodeling [5, 8–11]. Also, the adventitia has received more attention, and it was suggested that neovascularization of the adventitial vasa vasorum (VV) and local perivascular inflammation plays a key role in the development and progression of atherosclerotic plaques [12, 13]. The outcome of a recent systemic review and meta-analysis on CCTA plaque characterizations and major adverse cardiovascular events (MACE) [14] suggested that CCTA features of HRP can be a likely independent predictor of MACE, and proposed inclusion of CCTA evaluation of HRP in clinical practice [10, 14]. Sudden cardiac death (SCD) related to CAD is an extreme of MACE.

In *postmortem* practice, coronary calcifications as a sign of atherosclerotic progression can be detected easily by postmortem CT (PMCT). However, the presence of non-calcified plaque, coronary stenosis assessment, and most HRP characteristics can be evaluated only after visualization of the lumen of the vessel with a circulating contrast agent. Additionally, invasive postmortem angiographic techniques such as multi-phase postmortem computed tomograpghy angiography (MPMCTA) enable the evaluation of coronary artery lumen, stenosis, suspected occlusions, and above-mentioned characteristics of HRP [15–17].

Histopathological investigation of cross sections of coronary arteries is considered the “gold standard” postmortem method to investigate the heterogeneous composition of atherosclerotic plaques. The features of high-risk/vulnerable plaques (thin caps, large atheroma, inflammation) and the diverse parameters of so-called “acute plaques” (plaque rupture/erosion, mural thrombus, occluding thrombus, and thrombus age) are of relevance for the identification of culprit plaques in sudden deaths victims at autopsy [18]. Currently, how and to what extent the radiological markers of HRP as mentioned above relate to the histopathological features of acute plaques is unknown.

The goal of the study was to evaluate if clinically observed signs of so-called HRP can be observed in postmortem imaging at the level of the fatal plaque and to compare them to the histological findings.

## Materials and methods

### Case selection

**Fig. 1:**
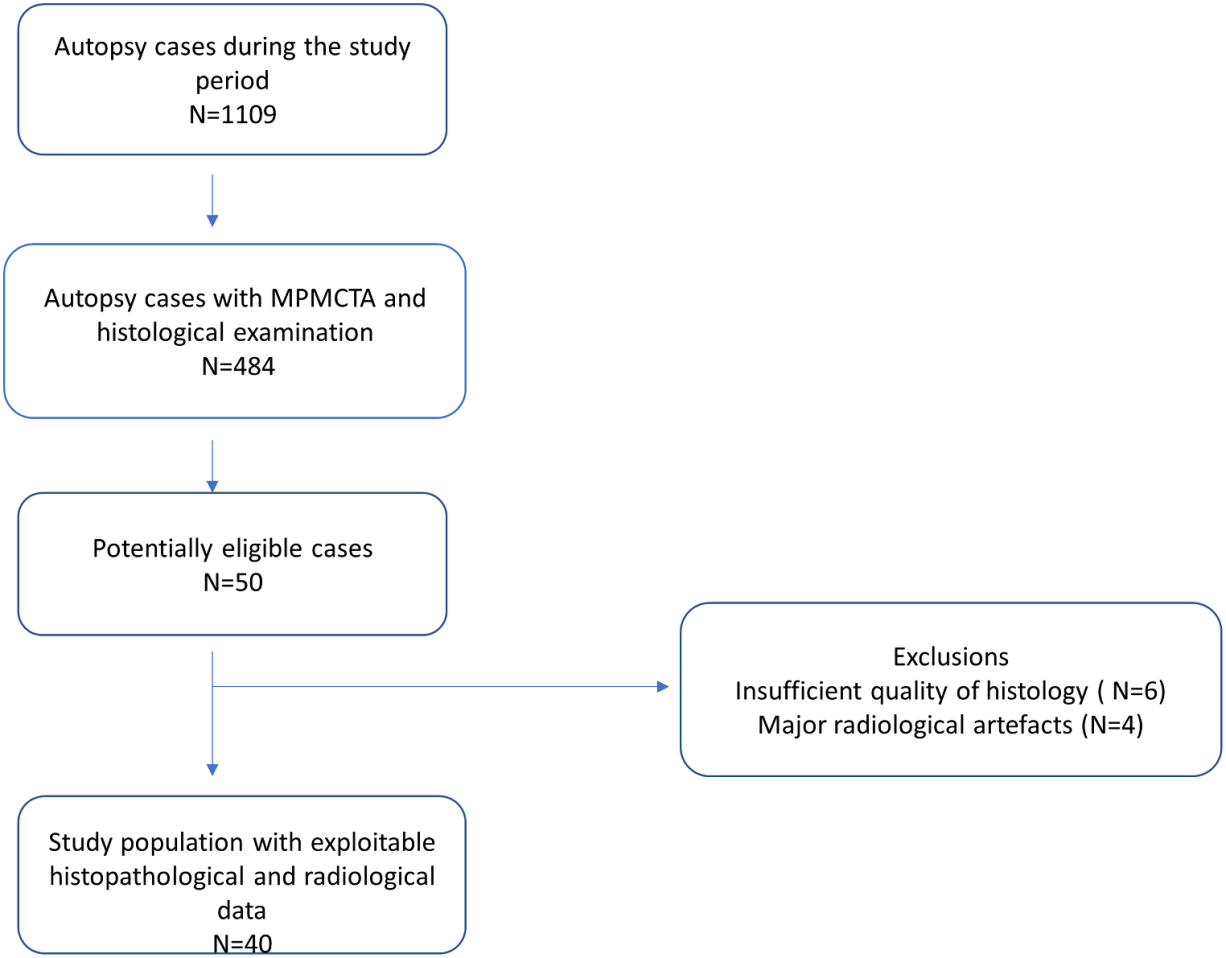
Study flow diagram

The study cohort consists of a series of autopsy cases of SCD victims due to acute coronary artery disease. Cases were collected from 2017 to 2020 and include all patients for whom both multi-phase postmortem CT angiography (MPMCTA) data and postmortem tissue blocks for further histopathological investigation of the coronary culprit lesion were available (see study flowchart in Fig. 1) [18, 19]. A full autopsy was performed on all cases according to the international guidelines [20] after an initial external examination and multi-phase postmortem CT angiography (MPMCTA) [21].

Clinical data, including age, gender, type and duration of symptoms, resuscitation attempts, and medication, were recorded. Data retrieved from the autopsy report were body weight, BMI, heart weight, topographic location of coronary occlusions in the arterial tree, coronary dominance, and results of toxicological analyses and postmortem serum troponin levels, if available.

Cases showing putrefaction, carbonization, traumatic lesions of the heart (not related to resuscitation attempts), and cases after percutaneous coronary revascularization procedures and/or coronary artery bypass grafting (type 4 and 5 of myocardial infarction) [22] were excluded. Cases where concomitant pathology or toxicology results could explain the death were excluded.

### Histopathological examination of coronary arteries

Archived segments of coronary arteries containing the culprit plaque (totally occluded or at least mural thrombosed lesion) during autopsy were collected for histological examination. For histopathological analysis of the occluded coronary artery, scanned hematoxylin and eosin (H&E) and trichrome-stained slides were reviewed. Two independent observers (with more than 10 years of experience in cardiovascular pathology) performed pathological evaluation. Consensus reading was obtained for the evaluation.

Coronary artery stenosis was graded on a 3-point scale as less than 50%, 50–75%, and more than 75%.

Calcifications of the plaque were evaluated considering their diameter and following the literature as without calcification, microcalcifications: 0.5–15 μm, punctuate/fragmented: 15 μm- 3 mm, sheet > 3 mm where both collagen matrix and necrotic core were calcified and nodular showing breaks in calcified plates with fragments of calcium separated by fibrin [23].

Regarding the composition of the plaque, plaques were classified as fibrous, fibrolipid or calcified. Additionally, the lipid core size was graded semiquantitatively as less than 10% lipids, 10–50% lipids, or more than 50% lipids of total plaque area.

Plaque complications were described as plaque rupture (disruption of a TCFA) with expulsion of the underlying necrotic core, clearly recognizable by the presence of cholesterol clefts), plaque erosion (thrombus adjacent to intact plaque surface with denuded endothelium), or a protruding calcified nodule (thrombi associated with eruptive, dense, calcific nodules) [3].

Age of the thrombus was categorized as fresh, subacute/ (lytic), or organized/old (organized).

Intraplaque inflammation was evaluated on a 2-point scale considering the percentage of area with inflammatory cells as none or with small foci (0–10%), moderate or severe (more than 10%).

Adventitial inflammation was graded on a 3-point ordinal scale as follows: 1, normal (scarce isolated cells); 2, inflammatory foci occupying less than 50% of the circumference of the artery, and the inflammatory zone’s thickness remaining smaller than the media’s thickness; 3, inflammatory foci occupying more than 50% of the arterial circumference or inflammatory zone’s thickness exceeding the media’s thickness.

Vasa vasorum extent was graded on a 3-point ordinal scale as follows: 1, normal; 2, increased, less than 50% of the arterial circumference; 3, markedly increased, more than 50% of the arterial circumference.

### Methods for the radiological evaluation

For all cases, a postmortem CT angiography was performed on a 64-row multidetector CT system (CT LightSpeed VCT, GE Healthcare) according to the standard protocol of the MPMCTA [21], including a noncontrast acquisition covering the entire body (from head to toe) followed by three angiographic phases, arterial, venous and dynamic. The same parameters were applied for each angiographic phase: helical acquisition from vertex to pelvis at 120 kV, 200–400 mA modulation, noise index 35, pitch 0,984:1, detector coverage 40 mm, slice thickness 1,25 mm, interval 0.625 mm, tube rotation 0.8 s, SFOV: 50 cm, and a standard algorithm of reconstruction, window width 400 (WW) and window level 40 (WL). For the arterial phase, a volume of 1200 ml of contrast agent, an oil-based solution consisting of a mixture of paraffin oil with 6% of contrast agent (Angiofil®, Fumedica), with a flow rate of 800 ml/min was injected via femoral artery cannulation. A volume of 1800 ml of the same contrast mixture was injected for the venous phase at a flow rate of 800 ml/min via femoral vein cannulation. For the dynamic phase, an additional 500 ml of contrast mixture was injected via the arterial system. During this phase, the filling was synchronized with acquisition and adapted in function of time of acquisition (time of injection 150 s) with a flow rate of 200 ml/min.

Coronary plaques in MPMCTA at the lesion level determined at autopsy [24] were analyzed after curved multiplanar (CMPR) and curvilinear reconstructions (CVR) using an advanced postprocessing software (Advantage Workstation, GE Healthcare) on a specific heart standard algorithm of reconstruction with a window width of 400 and a window level of 40, slice thickness of 0,625 mm with an interval of 0,312 mm, on the arterial and dynamic phases. The venous phase was not analyzed, considering it would not add further information.

The extent of coronary artery calcifications was evaluated using a semi-automated tool to calculate the coronary calcium score(CCS) on a specific unenhanced heart acquisition: cine rotation 0,9 s, detector coverage 20 mm, slice thickness 2,5 mm, acquisition of 8 images per 0,5 s, SFOV: 50 cm, 120 kV, 400 mA, DFOV: 25 cm, standard algorithm, no iterative reconstructions. The score was recorded globally and then separately for the involved vessel.

Additionally, we assessed the following parameters: lumen diameter stenosis, plaque enhancement, and the previously mentioned HRP characteristics consisting of remodeling index, NRS, low attenuation plaques, and SC.

NRS was defined on a cross-section of the coronary artery at the level of the culprit lesion as a thin ring-like hyperattenuating rim surrounding a low attenuating eccentric structure and was defined as present or absent.

Low attenuation plaque component (< 30 HU) was defined as the mean CT number within three regions of interest (approximately 0.5-1.0 mm^2^) randomly placed in the non-calcified portion of the plaque and was categorized as absent, <1 mm or ≥ 1 mm.

Spotty calcification was defined as a small, dense (> 130HU) plaque component surrounded by noncalcified plaque tissue, ≤ 3 mm in curved multiplanar reformat and was classified as absent, less than 1 mm and between 1 and 3 mm.

The degree of vessel stenosis was classified into three categories: <50%, between 50 and 75%, and > 75%. The remodeling index (RI) was calculated as the vessel cross-sectional area at the site of maximal stenosis divided by the average of proximal and distal reference segments’ cross-sectional areas [25]. A RI threshold of more or equal to 1.1 was considered for the definition of positive remodeling [9]. The measurement of minimal lumen area (MLA) was done on a curvilinear reconstruction in small axis of the vessel and calculated in mm^2^.

By simultaneously displaying noncontrast, arterial, and dynamic phases, the observers could determine plaque enhancement, which was considered positive when plaque exhibited visually higher attenuation in dynamic phase compared to native and arterial phases.

Image analysis was performed by two radiologists one with > 10 years of experience and 5 years of forensic radiology practice and a fellowship-trained cardiovascular radiologist with 8 years of experience. If consensus was not obtained, a senior cardiovascular radiologist with over 20 years of experience helped resolve the case.

### Statistical analyses

Statistical analyses were performed using STATA 16 software (*StataCorp. 2019. Stata Statistical Software: Release 16. College Station, TX: StataCorp LLC*).

Descriptive statistics of the study population’s characteristics and their radiological and histopathological data were reported as mean(sd) for continuous variables and as number(percent) for the categorical variables.

Correlations, which seemed pertinent considering the mechanism of coronary artery disease and literature data, were tested between radiological and histopathological findings using Fisher’s exact test to check the hypothesis that the rows and columns in a two-way table are independent.

The association between CAC score of the concerned vessel and the degree of histological calcification detected was performed by the Kruskall-Wallis equality-of-population rank test. However, the strength of the association between the global and culprit vessel CAC score was assessed using a robust regression and Spearman’s rho coefficient.

The agreement between stenosis of the lumen at histology and during radiological examination was assessed using the kappa-statistic measure of interrater agreement.

## Results

### Study population characteristics, autopsy and radiological findings

Out of the total number of 1109 autopsies performed during the study period, 50 cases fulfilled all inclusion criteria. After histological examination, 6 cases for which coronary thrombosis /culprit lesion was not confirmed (technical problems including bad decalcification) were excluded, and 4 cases after radiological examination with non-interpretable MPMCTA (one had layering artifacts, two had no identified culprit lesion on CT and one an excessive image noise due to obesity). Cases with only some minor non-evaluable features of the plaques on autopsy or MPMCTA, were not included. Finally, after excluding 10 above-mentioned cases for major histological or radiological artefacts, 40 cases were included (28 men and 12 women). The flow diagram for study cases is shown in Fig. 1. The characteristics of the study population, postmortem serum levels of troponins, and results of histological and radiological assessments are summarized in Tables 1, 2 and 3.

**Table 1:** Major characteristics of the study population (*N* = 40)

Female sex, N (%)	12 (30)
Age [years], mean (SD), min- max	53.3 (10.9), 29–78
Body mass index [kg/m^2^], mean (SD)	28.03 (6.7)
Heart weight [g], mean (SD)	445.5 (127.2)
Postmortem Hs-troponin [ng/L], median (IQR)	115 (154.5)
Symptoms	
No known, found dead home, N (%)	14 (35)
No known, witnessed collapse, N (%)	8 (20)
Symptoms within 24 h before the death, N (%)	18 (45)
Resuscitations attempts	
No, N (%)	17 (42.5)
Yes, N (%)	23 (57.5)

**Table 2:** Autopsy/histology findings at the level of fatal plaque

	N (%)
Localisation of the culprit lesion	
MS	1 (7.5)
AD	13 (32.5)
CX	3 (7.5)
RCA	23 (57.5)
Stenosis of the lumen	
Less than 50%	2 (5)
50–75%	10 (25)
more than 75%	28 (70)
Plaque complication, N of evaluated cases (*N* = 36)	
erosion	11 (30.6)
rupture	21 (58.3)
CTO	4 (11.1)
Age of the occlusion	
Fresh thrombus	27 (67.5)
Early organisation thrombus	5 (12.5)
Old thrombus	4 (10)
Very old (CTO)	4 (10)
Location of the plaque, N of evaluated cases (*N* = 34)	
Concentric	13 (38.24)
Excentric	21 (61.76)
Plaque composition, N of evaluated cases (*N* = 39)	
fibrous	2 (5)
fibrolipid with less than 10% of lipid	5 (12.5)
fibrolipid with 10–50% of lipid	8 (20)
fibrolipid with more than 50% of lipid	20 (50)
calcified	5 (12.5)
Calcifications, N of evaluated cases (*N* = 40)	
none	8 (20)
small	5 (12.5)
fragmented	11 (27.5)
sheets	14 (35)
nodules	2 (5)
Intraplaque inflammation, N of evaluated cases (*N* = 38)	
none or small foci, less than 10%	13 (34.2)
moderate or severe, more than 10%	25 (65.8)
Adventitial inflammation, N of evaluated cases (*N* = 39)	
None or isolated cells	8 (20.5)
Less than 50% of circumference of the artery with inflammatory zone less than 50% of the media	9 (23.1)
More than 50% of circumference of the artery or inflammatory zone more than 50% of the media	22 (56.4)
Vasa vasorum, N of evaluated cases (*N* = 39)	
Few VV (normal)	8 (20.5)
Dilated VV on less than 50% of the arterial circumference	13 (33.4)
Dilated VV on more than 50% of the arterial circumference	18 (46.1)

MS- main stem, LAD, left anterior descending artery, CX, circumflex artery, RCA, right coronary artery, CTO, chronic total occlusion, VV, vasa vasorum

**Table 3:** Radiological findings of the case and on the level of the fatal plaque

CAC score global, median (IQR)	314 (455)
CAC score of the vessel, median (IQR)	96 (312)
Remodeling index, mean (SD)	1.39 (0.71)
Stenosis of the lumen, N of evaluated cases (*N* = 39)	
Less than 50%, N (%)	2 (5.1)
50–75%, N (%)	5 (12.8)
More than 75%, N (%)	32 (82.1)
Minimal lumen area [mm^2^], mean (SD)	1.12 (1.22)
NRS	
No, N (%)	24 (60)
Yes, N (%)	16 (40)
Low attenuation plaque, N of evaluated cases (*N* = 39)	
No, N (%)	21 (53.9)
Yes, less than 1 mm, N (%)	6 (15.4)
Yes, more than 2 mm, N (%)	12 (30.7)
Spotty calcifications, N of evaluated cases (*N* = 39)	
No, N (%)	20 (51.3)
Yes, <1 mm, N (%)	11 (28.2)
Yes, 1–3 mm, N (%)	8 (20.5)
Enhancement of the plaque, N of evaluated cases (*N* = 36)	
No, N (%)	15 (41.7)
Yes, N (%)	21 (58.3)

### Correlations between radiological and histopathological findings

#### Coronary artery calcium (CAC) score

**Fig. 2:**
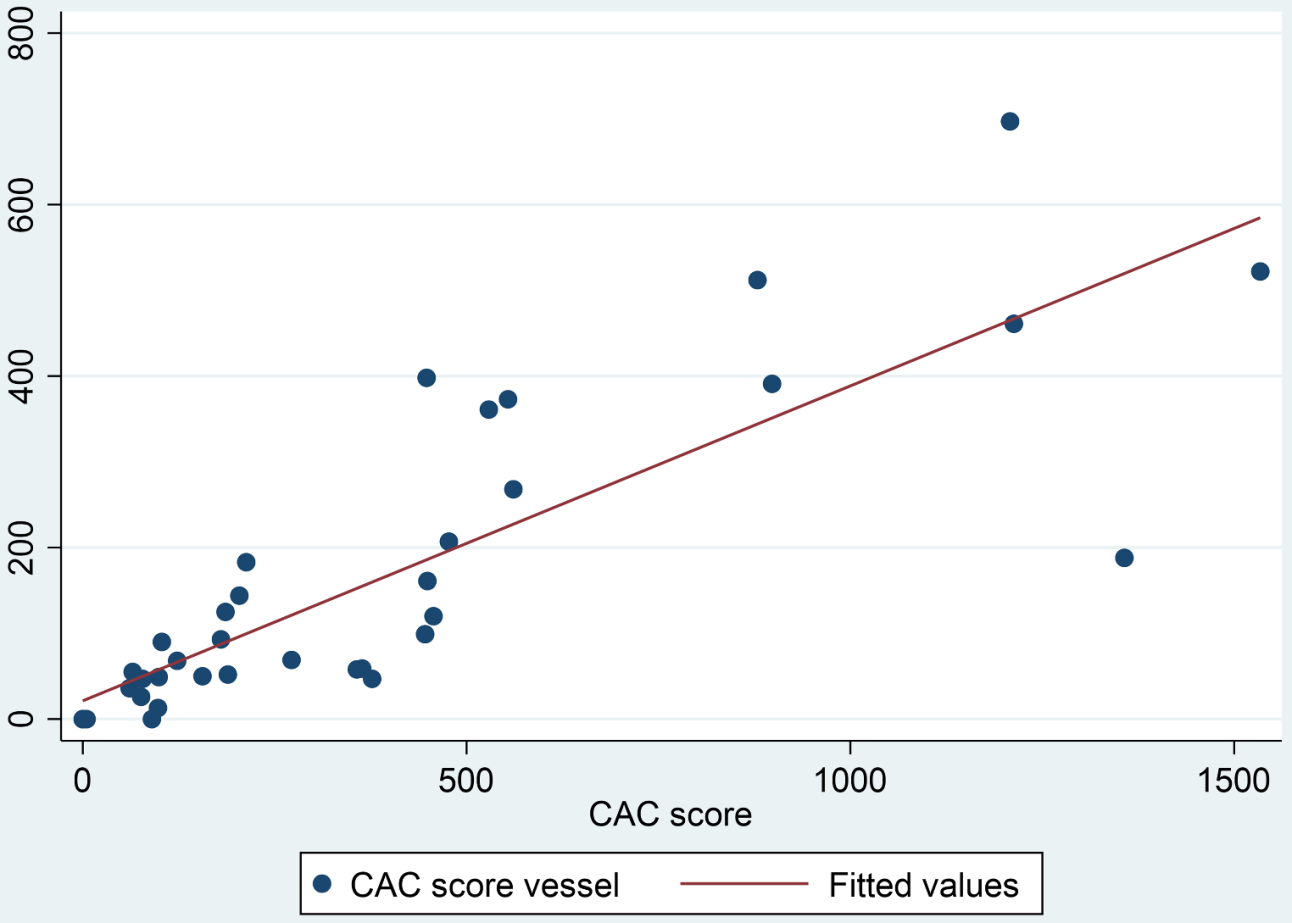
The correlation between CAC score global (x-axis) and for the concerned vessel (y-axis) was very strong; Spearman’s rho 0.91 p-value < 0.0001

There was a very strong correlation between global CAC score and the vessel with the culprit lesion. The calculated β-Coefficient using a robust regression model was β = 0.52 (p-value < 0.0001), and the Spearman’s rho correlation rho = 0.91 (p-value < 0.0001) (Fig. 2).

**Fig. 3:**
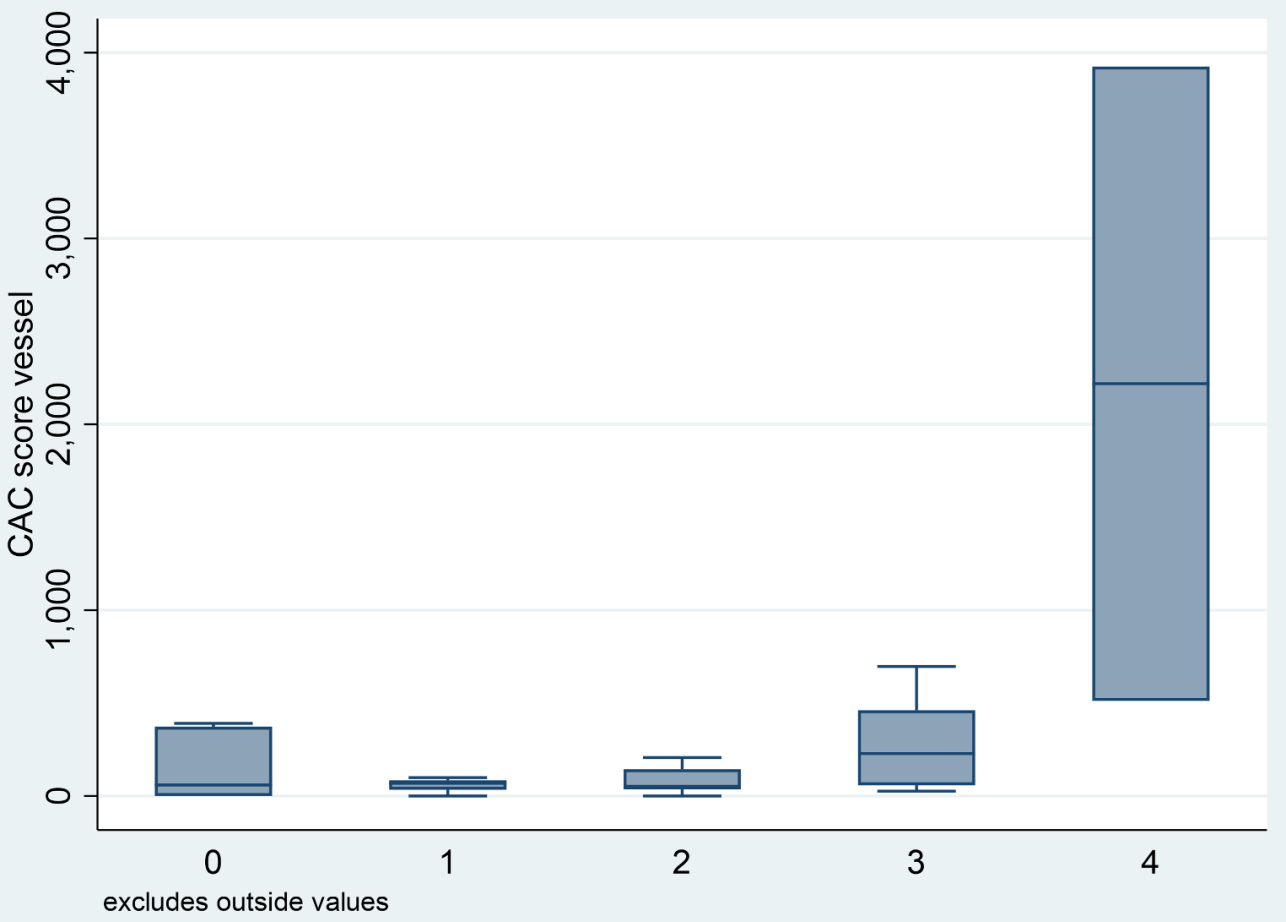
CAC scoring of the vessel versus histology of coronary calcifications; 0-no calcification; 1-micro; 2-fragmented; 3-sheet, 4-nodular Higher values of CAC score were observed for the histological group 3 and 4 (calcium sheet/ nodule). A statistically significant association was observed (*p* = 0.033)

The association between the CAC score of the concerned vessel and the degree of histologically detected calcification was performed by the Kruskall-Wallis equality-of-population rank test. A statistically significant association was observed (*p* = 0.033). Higher values of CAC score were observed for the histological groups 3 and 4 (respectively calcium sheet/ nodule) (Fig. 3).

#### Spotty calcifications (SC)

**Fig. 4:**
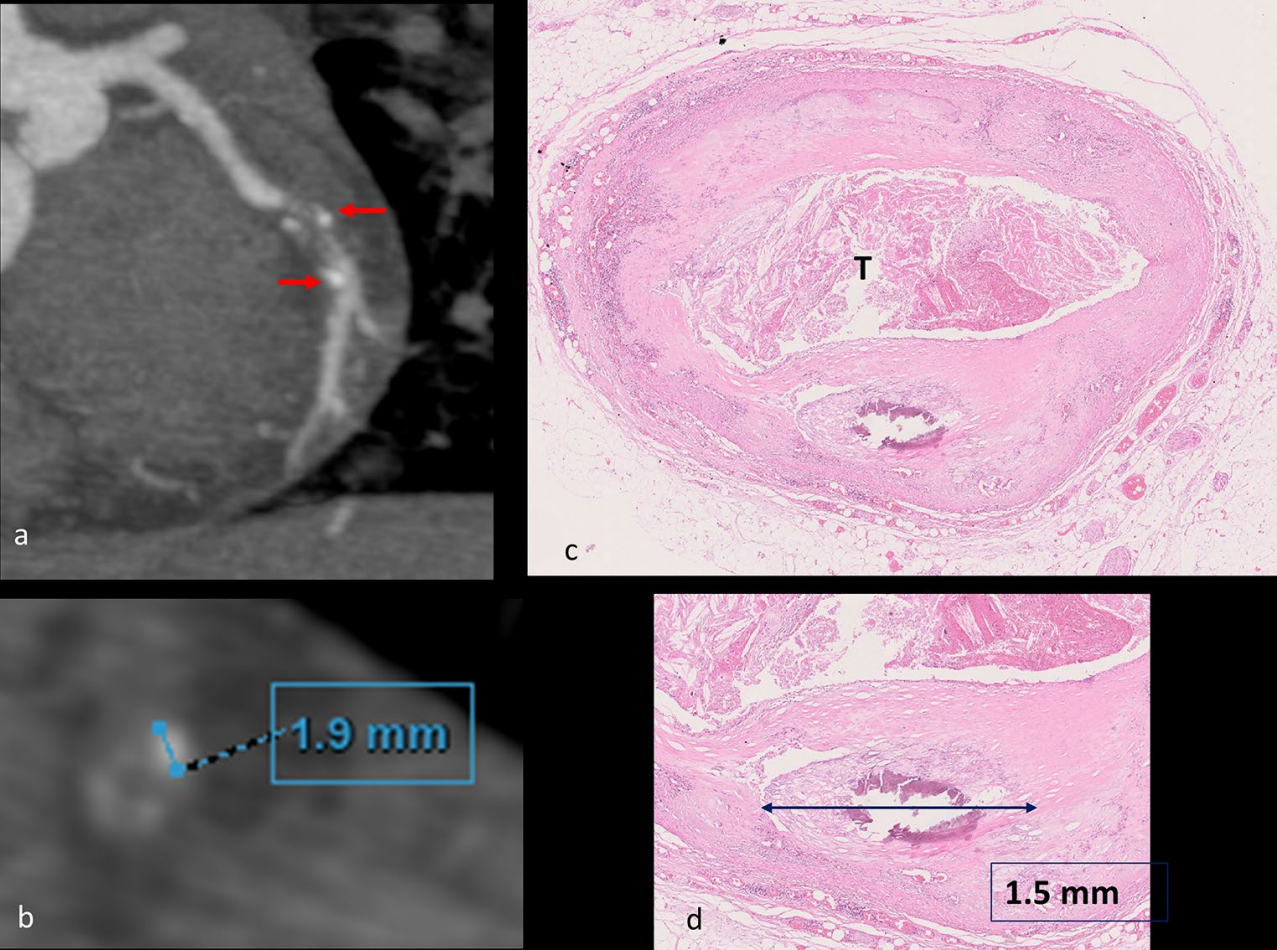
Spotty calcifications (red arrows) at radiological examination, corresponding to dense plaque component (> 130HU) measuring < 3 mm (**a**-**b**); curvilinear reconstruction of the CX artery, arterial phase of the MPMCTA (**a**); Cross section of the circumflex artery with measurement of the spotty calcification (**b**); histological slide of the CX artery stained with H&E showing a thrombotic occlusion (T) and a punctuate calcification of 1.5 mm of diameter (**c**, **d**)

A significant correlation (p-value 0.002) was observed between the presence of SC detected at radiological examination (Fig. 4) and the presence of punctuate/fragmented calcification at histology. There was no significant correlation between the presence of SC on MPMCTA for the type of plaque, type of thrombosis, and composition of the plaque.

#### Low attenuation plaque (LAP)

There was a non-significant difference for the presence of LAP on radiological examination and histological type of plaque, composition of the plaque, age of the thrombosis, and degree of calcifications, inflammation, and vasa vasorum. It should be noted that all cases with a lipid core consisting of over 50% of lipids histologically (considered as vulnerable plaques [3]) were visualized in more than 50% of PMCTA.

#### Napkin-ring sign (NRS)

**Fig. 5:**
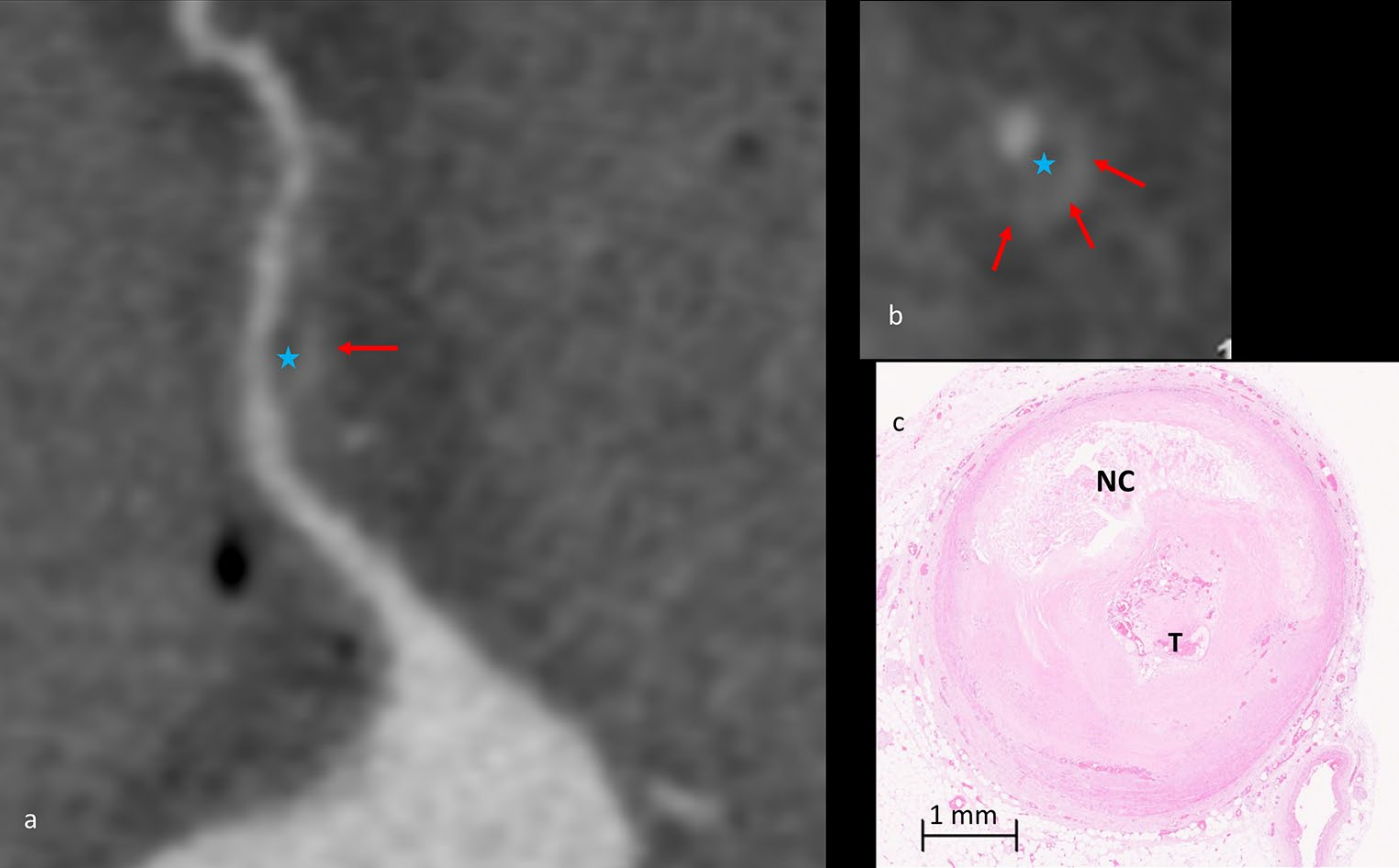
Napkin-ring sign (NRS) at MPMCTA; Curvilinear reconstruction of the right coronary artery (RCA), arterial phase of the MPMCTA (**a**) Cross section of the RCA, arterial phase of the MPMCTA (**b**) NRS seen at the level of the culprit lesion (red arrows), ring-like hyperattenuating rim surrounding a low attenuation eccentric structure (blue stars); Histological section of the RCA stained with H&E showing a recannalised thrombotic occlusion (T) and necrotic core riche in lipids (NC)

The NRS was observed in 40% of cases. There were significant correlations of the radiological presence of NRS (Fig. 5) for fibrolipidic composition of the plaque (p-value 0.007), severe intraplaque inflammation (p-value 0.017), severe adventitial inflammation (p-value 0.021) and an increased VV (p-value 0.012). No significant correlation was observed for the degree of calcification.

#### Remodeling index (RI)

**Fig. 6:**
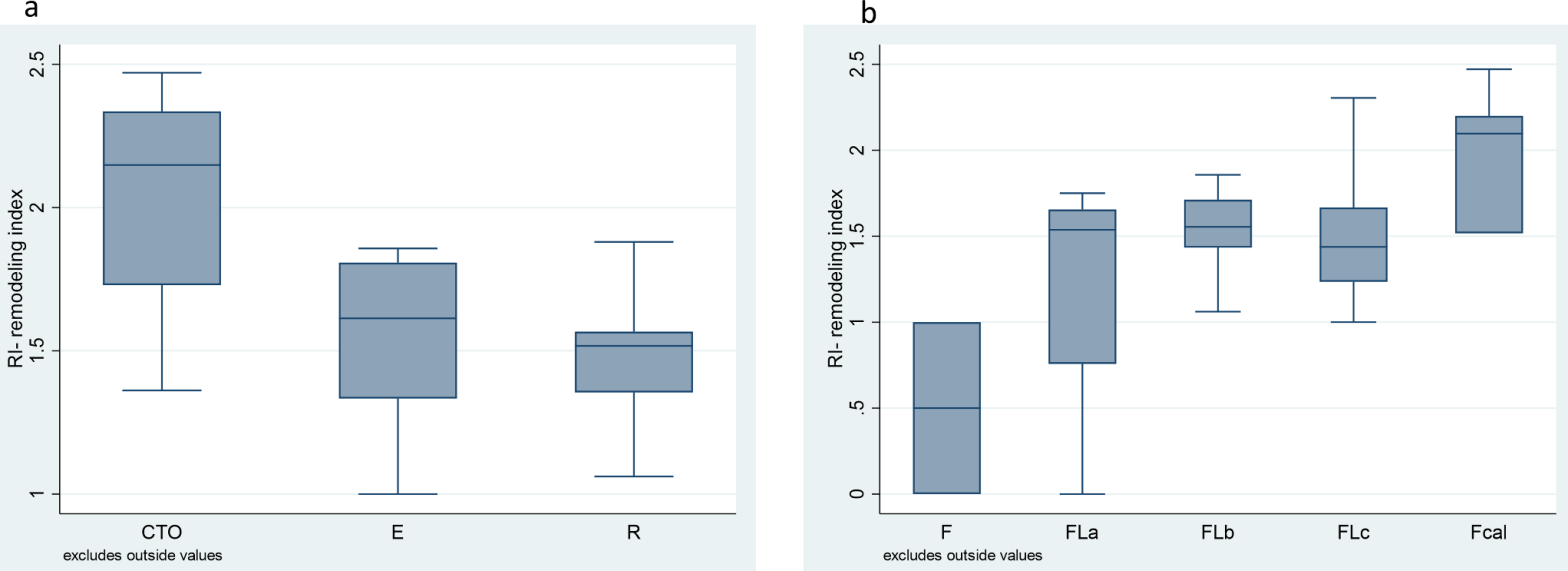
Remodelling index values in cases of chronic total occlusion (CTO), eroded (E) and ruptured (R) plaques (**a**) and in different types of plaques depending on their composition: fibrotic (f), fibrolipid with less than 10%, lipids (Fla), 10–50% lipids (Flb) or more than 50% lipids of total plaque area (Flc) and in calcified (Fcal) (**b**)

**Fig. 7:**
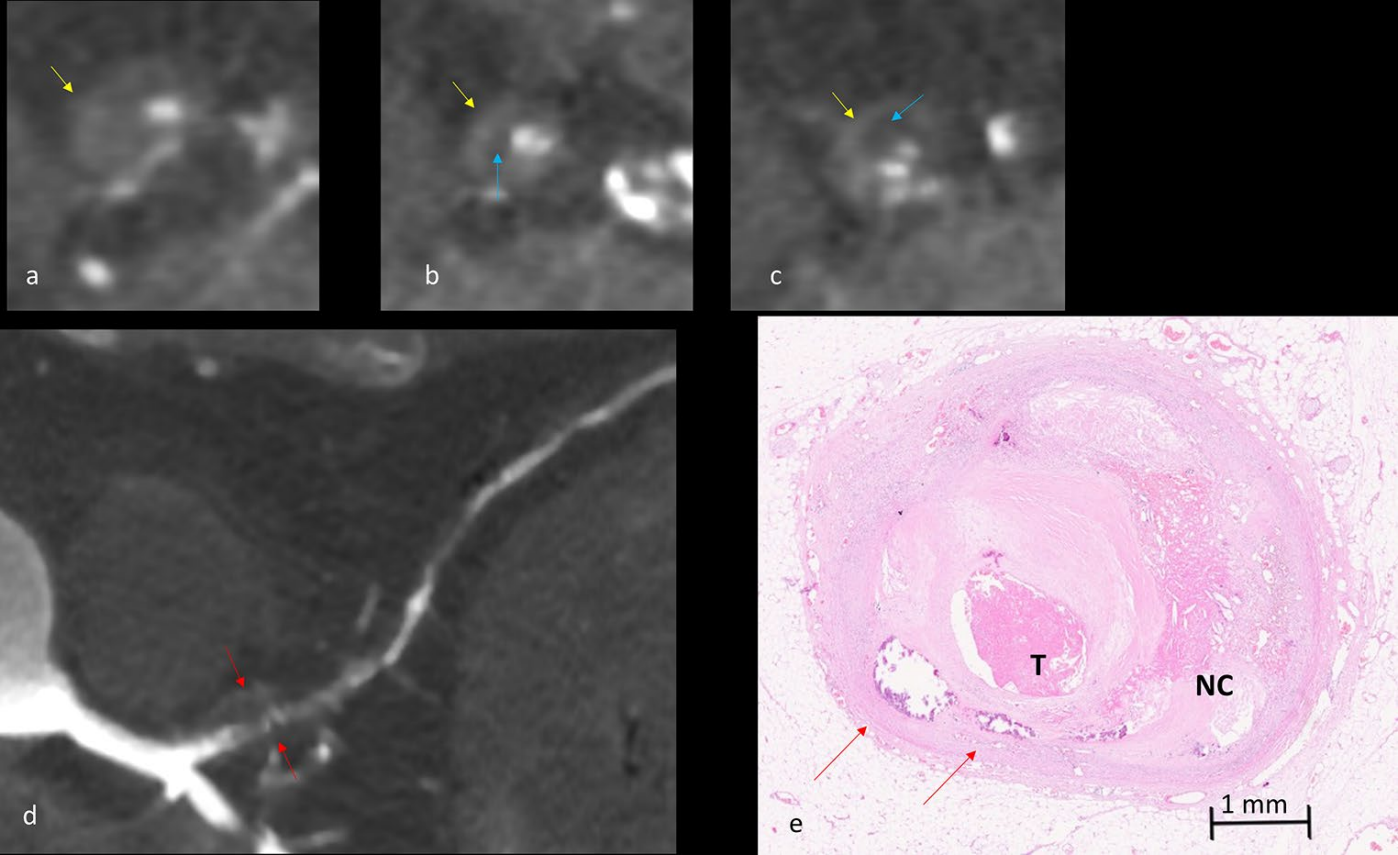
Positive remodeling and enhancement of the plaque at MPMCTA (**a**-**d**); Cross section of the left anterior descending artery (LAD), native phase of the PMCT (**a**); Cross section of the LAD, arterial phase of the MPMCTA (**b**); Cross section of the LAD, dynamic phase of the MPMCTA (**c**); Curvilinear reconstruction of the LAD, arterial phase of the MPMCTA (**d**); Plaque enhancement (yellow arrows), visually higher attenuation in dynamic phase compared to arterial phase; Low attenuation plaque component (blue arrows); Remodeling index (RI) (red arrows), here beeing 2,51, calculated as the vessel cross-sectional area at the site of maximal stenosis divided by the average of proximal and distal reference segments ‘cross-sectional areas. Histological slide of the LAD artery stained with H&E with thrombotic occlusion (T), calcifications (green arrows), lipids and hemorrhage in the necrotic core (NC)

A positive RI (≥ 1.1) was observed in 75% of cases. There was a significant difference (p-value 0.005) in RI for CTO versus an acute plaque complication (eroded and ruptured plaques). Regarding the composition of the plaque, RI was < 1 for fibrotic plaques and the highest values were observed for calcified plaques with a significant difference between groups (p-value 0.064) (Figs. 6 and 7). There was no significant correlation for plaque or adventitial inflammation and RI.

### Enhancement of the plaque

Enhancement of the plaque was noticed in 58.3% of cases (Table 3). Significant correlations for the presence of the enhancement of the plaque (Fig. 7) were observed for plaques with fibrolipidic composition (p-value 0.003) and a severe intraplaque inflammation (p-value 0.011). Enhancement of the plaque was not observed in fibrous and calcified plaques. There were no significant correlations between enhancement and other histological parameters.

### Stenosis

There was a poor agreement between stenosis of the lumen at histology at the plaque level and at radiological evaluation, this for all cases (58.97%, Kappa − 0.0833; expected agreement 62.13%) and for cases with stenosis over 75% (58.97%, Kappa-0.1387, expected agreement 53.97%).

## Discussion

In clinical radiology, various non-invasive cardiovascular imaging modalities have been applied to investigate the presence of coronary artery disease or its consequences by detecting coronary artery calcification, stenosis, or myocardial infarction, respectively. However, the main efforts of modern non-invasive imaging modalities are directed towards identifying asymptomatic non-obstructive vulnerable plaques by detecting signs of vulnerability as the presence of low-attenuation plaque, napkin-ring sign, spotty calcifications, and positive remodeling [5, 26]. In this study, we demonstrated that these signs can be detected at the level of fatal coronary occlusion in cases of SCD. We observed these signs in our series: SC in 49% of cases, LAP in 46% of cases, and NRS in 40%. In 58% of cases, we observed a similar type of plaque enhancement as was recognized previously in carotid arteries as a marker of plaque vulnerability closely related to ischemic stroke [27]. We recently reported that the latter correlates with histopathological signs of plaque inflammation, potentially serving as an additional imaging marker of plaque vulnerability [28]. Our findings are in line with case reports of suspected sudden coronary death, showing how CCTA can be employed to detect high-risk plaque features using histopathology as a gold standard [29].

In clinical practice, a RI threshold ≥ 1.1 visualized by CCTA was suggested to define positive remodeling [9]. In our series, we observed RI values over 1.1 in 75% of cases, and the mean RI was 1.39 ± 0.71. We also observed that the RI values were lower in cases with fibrotic plaques and higher for calcified plaques, which is compatible with postmortem and clinical studies [30–33].

The evaluation of coronary stenosis is considered a piece of critical autopsy information for the interpretation of CAD, as stenosis over 75% could be considered as a potential substrate for SCD in the absence of other causes [20]. We observed a poor agreement between stenosis of the lumen at histology at the plaque level and radiological evaluation. In the previous postmortem studies, Morgan et al. reported that there was an agreement between PMCTA and histological examination of the culprit lesion at autopsy, representing a critical stenosis of > 75%, with a sensitivity of 85.7% and specificity of 91.5% [15]. However, discrepancies between autopsy and PMCTA were reported when histology was assessed on a segmental basis, especially in regions of densely calcified vessels considered pathologically critical stenosis. Singh et al. investigated the sensitivity and specificity of PMCTA versus histopathology at autopsy in diagnosing coronary artery stenosis over 70% and observed a sensitivity of 61.5% and specificity of 91.7%. The authors reported a restrictive value of PMCTA in cases with pericardial hematoma and in stented coronary arteries, which were excluded from the study [34]. This indicates that stenosis of coronary arteries at imaging should be interpreted with caution.

Coronary artery calcifications are readily observed on PMCT and are considered a marker of atherosclerosis. However, it is well known that even zero or low CAC score on native PMCT cannot exclude the presence of myocardial infarction related to ACAD. This paradoxical discrepancy between imaging and autopsy findings can be explained by considering the pathophysiology of atherosclerosis and coronary thrombosis resulting in SCD, especially in young patients [35–40]. In our series, the median coronary artery calcium score was 314, corresponding to the “moderately to severely increased risk” group in clinical practice. However, there was probably some bias in case selection to perform PMCTA as cases without calcium in native PMCT had no systematic postmortem angiography.

In conclusion, clinically observed signs of so-called HRP can be observed in postmortem imaging at the level of the fatal plaque in the majority of cases. Moreover, an enhancement of the plaque could be considered as a new marker of inflammation, which could have some clinical role in risk evaluation. These signs could be considered as additional signs of vulnerability of the coronary plaque while interpreting PMCTA and could be hopefully useful to develop the prediction model to differentiate or diagnose sudden coronary cardiac death at forensic autopsies. More studies are needed to confirm and further our knowledge of these signs in postmortem radiological practice.

## Limitations of the study

The study’s major limitation is its retrospective and forensic nature. Some of the collected coronary arteries were excluded as they presented technical problems mostly related to the absence or insufficient decalcification.

## Data Availability

The data supporting the findings of this study are available upon request. For inquiries regarding data access or any additional information related to the dataset, please contact the corresponding author.

## Abbreviations

CAD: coronary artery disease
CCTA: coronary computed tomography angiography
CMPR: curved multiplanar recontruction
CVR: curvilinear reconstruction
CTO: chronic total occlusions
HRP: high-risk plaque
HU: Hounsfield unit
IHD: ischemic heart disease
MACE: major adverse cardiovascular events
MLA: minimal lumen area
MPMCTA: multi-phase postmortem computed tomography angiography
NRS: napkin-ring sign
PMCTA: postmortem computed tomography angiography
PR: positive remodeling
SCD: sudden cardiac death
TCFA: thin-cap fibroatheroma
VV: vasa vasorum
